# Tumor-infiltrating lymphocytes as a predictor of axillary and primary tumor pathological response after neoadjuvant chemotherapy in patients with breast cancer: a retrospective cohort study

**DOI:** 10.1007/s10549-024-07334-6

**Published:** 2024-05-04

**Authors:** Kian Chin, Amalia H. Landén, Anikó Kovács, Fredrik Wärnberg, Maria Ekholm, Per Karlsson, Roger Olofsson Bagge

**Affiliations:** 1 Institute of Clinical Sciences, Sahlgrenska Academy at Gothenburg University, Gothenburg, Sweden; 2 Department of Clinical Pathology, Sahlgrenska University Hospital, Gothenburg, Sweden; 3 Department of Oncology, Institute of Clinical Sciences in Sahlgrenska Academy, University of Gothenburg, Sahlgrenska University Hospital, Gothenburg, Sweden; 4 Department of Oncology, Ryhov County Hospital, Jönköping, Sweden; 5 Department of Biomedical and Clinical Sciences, Division of Oncology, Linköping University, Linköping, Sweden; 6 Department of Surgery, Sahlgrenska University Hospital, Region Västra Götaland, Gothenburg, Sweden

**Keywords:** Breast cancer, Neoadjuvant chemotherapy, Tumor-infiltrating lymphocytes, Axillary lymph node dissection, Tumor response

## Abstract

**Purpose:**

Tumor-infiltrating lymphocytes (TILs) can predict complete pathological response (pCR) of tumor in the breast but not so well-defined in the axilla after neoadjuvant chemotherapy. Since axillary surgery is being increasingly de-escalated after NACT, we aimed to investigate the relationship between TILs and pCR in the axilla and breast, as well as survival amongst NACT patients.

**Methods:**

Clinicopathological data on patients who underwent NACT between 2013 and 2020 were retrospectively examined. Specifically, pre-TILs (before NACT), post-TILs (after NACT) and ΔTIL (changes in TILs) were assessed. Primary endpoint was pCR and secondary endpoints were breast cancer-free interval (BCFI) and overall survival (OS).

**Results:**

Two hundred and twenty patients with nodal metastases were included. Overall axillary and breast pCR rates were 42.7% (94/220) and 39.1% (86/220), respectively, whereas the combined pCR rate was 32.7% (72/220). High pre-TILs (OR 2.03, 95% CI 1.02–4.05; *p *= 0.04) predicted axillary pCR whereas, high post-TILs (OR 0.33, 95% CI 0.14–0.76; *p *= 0.009) and increased ΔTILs (OR 0.25, 95% CI 0.08–0.79; *p *= 0.02) predicted non-axillary pCR. TILs were not a significant predictor for BCFI and OS.

**Conclusions:**

This study supports the potential use of pre-TILs to select initially node-positive patients for axillary surgical de-escalation after NACT.

## Introduction

Historically, surgery has been the primary treatment for breast cancer [1]. The realization of the benefits of adjuvant endocrine therapy and chemotherapy [1] in the 1980s marked the beginning of the modern era of breast cancer treatment. The recent approach with NACT has provided further advancement in breast cancer treatment. In the past decade, NACT has evolved from an option for locally advanced cancers to a standard curative modality for up to 60% of patients with human epidermal growth factor receptor-2 (HER2) positive and triple-negative breast cancer (TNBC) [2]. However, the increased use of NACT has also presented new challenges in surgery for example the accuracy in axillary staging and treatment options for residual tumors.

Following NACT, pCR can occur up to 66% of patients with certain tumor subtypes [3]. Specifically, among patients with initial axillary node metastases, pCR rates have been reported to range from 35 to 63% [4]. However, these high rates of axillary pCR have led to discussions on how to accurately stage the axilla after NACT. The practice of sentinel lymph node biopsy (SLNB) or targeted axillary dissection (TAD) after NACT for patients with nodal metastases is sporadic and variable amongst surgeons due to the uncertainties in these methods. Several trials have shown that post-NACT SLNB was associated with false negative rates ranging from 12.6 to 14.2% [5–8], which have sparked debates on the safety of avoiding lymph node clearance after NACT.

The interaction between cancer cells and the immune system is complex and dynamic [9]. This interplay may partly be represented by the presence of tumor-infiltrating lymphocytes (TILs) in the tumors. An elevated immune infiltrate with diverse T-cell, with predominance of CD8 + T-cells, have been associated with improved survival outcomes [10], especially in patients with HER2-positive disease. During NACT, there is evidence that suggest the immune microenvironment surrounding the tumor is altered by the treatment [11], which raises new surgical dilemmas. For example, changes in the amount of stromal TILs after NACT have been shown to be associated with pCR rates and, subsequently, also survival outcomes [12, 13]. Since axillary surgery after NACT is a topic of contentious discussion, utilizing an immune microenvironment factor like TILs as a potential predictor of axillary pCR, would be highly relevant in individualizing patient treatment in de-escalating axillary surgery. However, currently the clinical evidence for TILs to be considered as a mainstream predictor of axillary pCR [14] is limited.

This study aimed to investigate the relationship between TILs in the primary breast tumor and the pathological response in both the axilla and breast after NACT. The secondary aim was to study TILs as a prognostic factor for breast cancer-free interval (BCFI) and overall survival (OS).

## Methods and materials

### Patients and clinical data

**Fig. 1:**
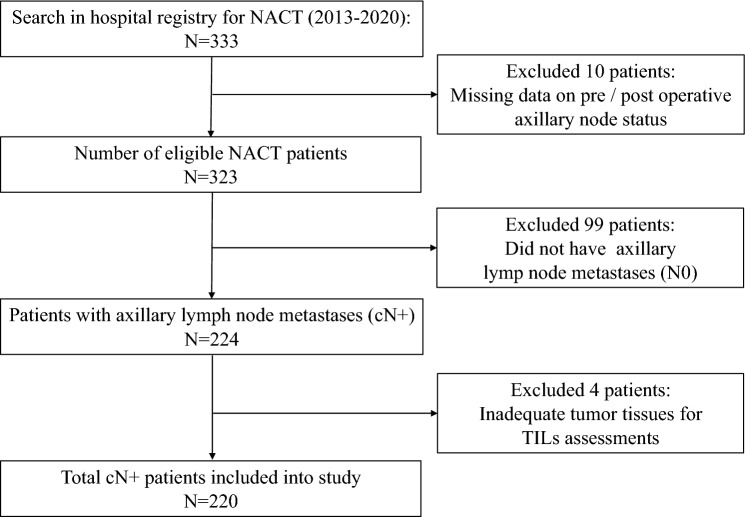
Flow chart showing the process of patient inclusion and exclusion for the study period of 2013–2020. First exclusion (*n *= 10) was based on patients with no available recorded data on axillary status or did not undergo axillary surgery due to distant metastases at diagnosis. Second exclusion (*n *= 99) was based on patients without axillary metastases. Third exclusion was based on inadequate archived breast cancer tissues to conduct TILs assessment. *NACT* neoadjuvant chemotherapy, *TILs* tumor-infiltrating lymphocytes

All patients with invasive breast cancer who underwent NACT at Sahlgrenska University Hospital in Sweden between 2013 and 2020 were identified from the hospital’s registry. Patients with all tumor sizes, axillary lymph node metastases (cN +) but without distant metastases (stages I to III) were included in the study. Axillary nodal metastasis was confirmed based on clinical suspicion via palpation, ultrasound assessment only or pathological examination of biopsied tissues. Patients who subsequently developed distant metastases during NACT did not undergo surgery and were therefore excluded from the study as the axillary nodal status after NACT were not available for analyses (Fig. 1). Data related to patients' clinical care, tumor biology (pre- and postoperative), NACT, surgery, adjuvant treatment, recurrences and survival were collected by a retrospective review of medical records. As NACT treatment protocols have evolved over time, the cohort characteristics were compared over two treatment periods, determined on a pragmatic basis (2013–2016 and 2017–2020), to identify any potential cohort differences.

### Materials

Histological slides of formalin-fixed, paraffin-embedded tumor tissues stained with hematoxylin and eosin (H&E) were retrieved from the archive. The amount of TILs in the archived breast tissue specimens were reviewed by a board-certified and subspecialized breast histopathologist (AK).

### Evaluation of TILs

According to the guidelines of the International Immuno-Oncology Biomarker Working Group, TILs assessments can be based on the amount found in the tumor stroma of the breast. However, TILs in lymph nodes were not evaluated as lymphocytes are naturally present in abundance making it technically difficult to quantify for TILs. In this study, stromal TILs were denoted as TILs found in the breast stromal tissues from preoperative core biopsies and surgical specimens. Tumor-infiltrating lymphocytes were defined as the percentages of stromal areas in the breast cancer occupied by mononuclear inflammatory cells. The assessment of TILs was conducted in both pre-NACT and post-NACT tumor tissues, using semicontinuous variables: less than 1, 1 to 9%, 10 to 49%, 50 to 74%, and 75% or greater. A predefined cutoff of 10% was used to classify TILs as either low (< 10%) or high (≥ 10%) [15, 16]. TILs found in tumor tissues before and after NACT were referred to as pre-TILs and post-TILs, respectively. Changes in amount of TILs after NACT were denoted as ΔTILs differentials and categorized as follows: ΔTILs^no change^ (from low to low or high to high TILs), ΔTILs^increase^ (from low to high TILs), and ΔTILs^decrease^ (from high to low TILs). Specifically, those patients with pCR, with no residual invasive tumor cells and absence of any post-TILs, were designated into the low TILs group in subsequent statistical analyses. Whereas pCR with evaluable TILs were designated accordingly as described above.

### Clinical data definitions and classifications

A cut-off of ≥ 10% was used to define estrogen receptor (ER) and ≥ 20% for progesterone receptor (PR) positivity. Ki-67 expression was categorized into three groups: low (≤ 5%), intermediate (6–29%), and high (≥ 30%). All ER, PR, tumor grades and Ki-67 expressions were assessed according to the Swedish National Guidelines versions 2013–2020 [17], where global score in Ki-67 was used. Human epidermal growth factor (HER2) receptor positivity was confirmed by immunohistochemistry and silver in-situ hybridization for patients with HercepTest scores (2 +). Due to the small proportion of grade 1 tumors, they were grouped with grade 2 and tumors were dichotomized into low (grades 1 & 2) and high (grade 3).

Tumor subtypes were classified based on immunohistochemical staining (IHC) and in-situ hybridization according to the Swedish National Guidelines as follows [18]: luminal A (ER + and PR + , HER2-, grade 1 or 2, and Ki-67 low or intermediate), luminal B HER2- (ER + and PR-, HER2-, grade 2 or 3, and Ki-67 intermediate or high), HER2 + luminal (ER + and/or PR + and HER2 +), HER2 + non-luminal (ER and PR-, HER2 +), and TNBC (ER and PR-, HER2-). Due to the small number of luminal A cancers, they were grouped together with luminal B and denoted as luminal cancers in the logistic regression analyses. The Miller Payne five-point grading system (MPG) was used to grade tumor responses in the breasts only and dichotomized as pathological complete response (pCR) (MPG grade 5) vs. non-pCR (MPG grades 1–4) [19]. Tumor pathological responses were categorized into 3 groups: response in the axilla, breast, or both (combined). All pCRs in the axilla and breast were defined as present irrespective of whether there were pCR in the other sites namely breast or axilla, respectively.

Time to locoregional and distant recurrences as well as survival was calculated from the date of diagnosis with a last follow-up date of October 31, 2022. The definitions of BCFI and OS according to the Standardized Definitions for Efficacy End Points (STEEP) in Adjuvant Breast Cancer Clinical Trials [20] were used in this study.

### Systemic and local treatments

All patients received treatments according to the Swedish National Breast Cancer Treatment Guidelines from 2013 to 2020 [17, 18, 21]. Neoadjuvant chemotherapy consisted of at least six cycles of adequately dosed chemotherapy, typically starting with anthracyclines and taxanes. A standard regimen included three doses of epirubicin (75–90 mg/m^2^) and cyclophosphamide (600 mg/m^2^), followed by three doses of docetaxel (75–100 mg/m^2^) or 9–12 cycles of weekly paclitaxel (80 mg/m^2^). A few patients also received carboplatin in addition to paclitaxel in line with research treatment protocols for patients with TNBC. Patients with HER2-positive breast cancer were treated with a combination of chemotherapy and trastuzumab (before 2015) or dual anti-HER2 blockade with trastuzumab and pertuzumab (from 2015 onwards). Completion of NACT was pragmatically defined as when patients had received at least 75% of their planned treatment doses, without considering the dose-intensity factor. Non-completion of NACT was defined when treatments were stopped prematurely due to severe side effects, toxicity, or disease progression, regardless of the received doses of NACT. No further adjustments were made in the study analyses for the non-NACT completion group as this is a heterogeneous group with limited retrospective clinical information to allow accurate and statistically meaningful analyses. Axillary metastases were treated with lymph node dissection (ALND) followed by locoregional radiotherapy, regardless of tumor response after NACT.

### Statistical analysis

Chi-squared test was used to report and compare pCR rates. Multivariable logistic regressions were performed to compare the relationship of TILs with axillary, breast, combined pCR rates and other clinic-histopathological parameters. Odds ratios (OR) and their 95% confidence intervals (CI) were reported. Sensitivity, specificity, positive (PPV) and negative (NPV) predictive values were analyzed as performance characteristics for TILs in predicting axillary status after NACT. For BCFI and OS, Kaplan–Meier curves were plotted and compared using the log-rank test.: The Cox proportional hazard model was used to report hazard ratios (HR) with 95% CI. The level of statistical significance was set at *p *< 0.05. All statistical analyses were performed using IBM SPSS Statistics version 29.0.0.0 (241), licensed in 2022.

## Results

### Patient characteristics

A total of 220 patients with axillary metastases who underwent NACT were included in the study. Over time, statistically significant increases were observed in the proportion of NACT patients with unifocal cancers (78 to 90%, *p *= 0.01) and grade 3 cancers (28 to 47%, *p *= 0.007). Otherwise, there were no statistically significant differences in the cohort characteristics between the two treatment periods (Table 1). Of note, amongst the high TILs group, 96% (64/66) and 76% (35/46) of patients had pre-TIL^high^ and post-TILs^high^ within the 10–49% category, respectively with only few allocated into the higher TILs category (over 50%).

**Table 1:** This showed overall cohort and tumor characteristics for the whole study period 2013–2020

	NACT treatment periods						*p*-value^a^
	Whole period		2013–2016		2017–2020		
	N	(%)	N	(%)	N	(%)	
Age (years)							
< 50	105	(48)	40	(47)	65	(49)	0.77
≥ 50	115	(52)	46	(53)	69	(51)	
Menopause status (cut-off age 50)							
Pre	129	(59)	48	(56)	81	(60)	0.50
Post	91	(41)	38	(44)	53	(40)	
Body mass index (kg/m^2^)							
< 30	179	(81)	72	(84)	107	(80)	0.47
≥ 30	41	(19)	14	(16)	27	(20)	
Type of cancer^b^							
Invasive ductal	196	(89)	71	(83)	125	(93)	0.05
Invasive lobular	19	(9)	12	(14)	7	(5)	
Mixed type	5	(2)	3	(3)	2	(2)	
Extent of cancer in the breast^b^							
Unifocal	188	(86)	67	(78)	121	(90)	0.01
Multifocal	31	(14)	19	(22)	13	(10)	
Tumor size (TNM system)^b^							
T1: < 2 cm	36	(16)	12	(14)	24	(18)	0.89
T2: ≥ 2-5 cm	115	(52)	46	(53)	69	(51)	
T3: > 5 cm	61	(28)	25	(29)	36	(27)	
T4: Any size^c^	8	(4)	3	(4)	5	(4)	
Tumor grade^b^							
Low (1&2)	132	(61)	61	(72)	71	(53)	0.007
High (3)	86	(39)	24	(28)	62	(47)	
Pre-TILs^b^							
Low (< 10%)	153	(70)	59	(69)	94	(70)	0.81
High (≥ 10%)	67	(30)	27	(31)	40	(30)	
Tumor subtypes^b^							
Luminal A and B	77	(35)	37	(43)	40	(30)	0.15
HER2 + /Luminal	50	(23)	19	(22)	31	(23)	
HER2 + /non-Luminal	49	(22)	18	(21)	31	(23)	
Triple negative	44	(20)	12	(14)	32	(24)	
Axillary pCR							
No	126	(57)	52	(60)	74	(55)	0.44
Yes	94	(43)	34	(40)	60	(45)	
Breast pCR							
No	134	(61)	58	(67)	76	(57)	0.11
Yes	86	(39)	28	(33)	58	(43)	
Combined pCR (Breast & Axilla)							
No	148	(67)	63	(73)	85	(63)	0.13
Yes	72	(33)	23	(27)	49	(37)	
NACT completion^d^							
No (< 75%)	23	(10)	8	(9)	15	(11)	0.66
Yes (≥ 75%)	197	(90)	78	(91)	119	(89)	

In addition, comparison between two arbitrary treatment periods of 2013–2016 and 2017–2020 was also conducted to identify possible differences in cohort characteristics as they were changes in national treatment guidelines for neoadjuvant chemotherapy over the study period

^a^NACT completion defined as patient received ≥ 75% of the prescribed intended treatments

^b^Denoted pre-NACT status

^c^Presence of skin ulceration or invasion of underlying structures like muscles or ribs irrespective of the size of the index breast cancer.

^d^Chi-square test with *p *< 0.05 is defined as statistically significant

*HER2* + human epidermal growth factor receptor-2, *NACT* neoadjuvant chemotherapy, *pCR* complete pathological response, *TNM system* staging with primary tumor, regional nodes and distant metastasis of breast cancer staging system, *pre-TILs* tumor-infiltrating lymphocytes present in breast cancer before start of neoadjuvant chemotherapy

Axillary metastases were diagnosed through fine needle aspiration in 74% (162/220), core biopsy in 14% (*n *= 30/220), SLNB before NACT in 8% (17/220), and clinical examination only in 5% (11/220) of the patients. Amongst the eleven patients with suspicious lymph nodes based on clinical examinations, seven were found to have axillary metastases after NACT. For the four patients, whose axillary metastases were not histopathologically detected after NACT, three had no evidence of regression of previously suspected metastases whereas one had lymph nodes with evidence of tumor regression.

### TILs in relation to pathological tumor responses

Proportions of patients with tumors containing TILs^low^ and TILs^high^ were compared with the respective pCR rates in the axilla, breast, and combined (Table 2).

**Table 2:** Table showed associations of pre-TILs, post-TILs and ΔTILs with the of axillary, breast and combined (both breast and axilla) pCR status after neoadjuvant chemotherapy

	*n*	Axillary pCR		Breast pCR		Combined pCR	
		*n* (%)	*p*-value^a^	*n* (%)	*p*-value^a^	*n* (%)	*p*-value^a^
*Pre-TILs*							
LOW (< 10%)	153	55 (35.9)	0.002	48 (31.4)	< 0.001	39 (25.5)	< 0.001
Luminal A&B	62	9 (14.5)		4 (6.4)		4 (6.4)	
HER2 + / Luminal	37	13 (35.1)		15 (40.5)		11 (29.7)	
HER2 + / non-Luminal	30	22 (73.3)		23 (76.6)		19 63.3)	
Triple Negative	24	11 (45.8)		6 (25)		5 (20.8)	
HIGH (≥ 10%)	67	39 (58.2)		38 (56.7)		33 (49.3)	
Luminal A&B	15	3 (20.0)		2 (13.3)		2 (13.3)	
HER2 + / Luminal	13	8 (61.5)		10 (76.9)		7 (53.8)	
HER2 + / non-Luminal	19	18 (94.7)		15 (78.9)		15 (78.9)	
Triple Negative	20	10 (50.0)		11 (55)		9 (45.0)	
*Post-TILs*							
LOW (< 10%)	174	82 (47.1)	0.01	78 (44.8)	< 0.001	66 (37.9)	0.001
Luminal A&B	63	11 (17.5)		5 (7.9)		5 (7.9)	
HER2 + / Luminal	39	19 (48.7)		25 (64.1)		18 (46.2)	
HER2 + / non-Luminal	42	35 (83.3)		33 (78.6)		30 (71.4)	
Triple Negative	30	17 (56.7)		15 (50.0)		13 (43.3)	
HIGH (≥ 10%)	46	12 (26.1)		8 (17.4)		6 (13.0)	
Luminal A&B	14	1 (7.1)		1 (7.1)		1 (7.1)	
HER2 + / Lum	11	2 (18.2)		0 (0.0)		0 (0.0)	
HER2 + / non-Lum	7	5 (71.4)		5 (71.4)		4 (57.1)	
Triple Negative	14	4 (28.6)		2 (14.3)		1 (7.1)	
*Δ TILs*							
NO CHANGE	143	57 (39.9)	< 0.001	48 (33.6)	< 0.001	41 (28.7)	< 0.001
Luminal A&B	60	10 (16.7)		5 (8.3)		5 (8.3)	
HER2 + / Luminal	30	13 (43.3)		15 (50.0)		11 (36.7)	
HER2 + / non-Luminal	29	23 (79.3)		22 (75.9)		19 (65.5)	
Triple Negative	24	11 (45.8)		6 (25.0)		6 (25.0)	
INCREASE	28	5 (17.9)		4 (14.3)		2 (7.1)	
Luminal A&B	8	0 (0.0)		0 (0.0)		0 (0.0)	
HER2 + / Luminal	9	1 (11.1)		0 (0.0)		0 (0.0)	
HER2 + / non-Luminal	4	2 (50.0)		3 (75.0)		2 (50.0)	
Triple Negative	7	2 (28.6)		1 (14.3)		0 (0.0)	
DECREASE	49	32 (65.3)		34 (69.4)		29 (59.2)	
Luminal A&B	9	2 (22.2)		1 (11.1)		1 (11.1)	
HER2 + / Luminal	11	7 (63.6)		10 (90.9)		7 (63.6)	
HER2 + / non-Luminal	16	15 (93.8)		13 (81.3)		13 (81.3)	
Triple Negative	13	8 (61.5)		10 (76.9)		8 (61.5)	

These associations according to tumor subtypes were also analyzed. Cut-off between low and high TILs was 10%

^a^Cross tabulations analyses with chi-square test, *p *< 0.05 defined as statistically significant

*pCR* complete pathological response, *pre-TILs* tumor-infiltrating lymphocytes before neoadjuvant chemotherapy, *post-TILS* tumor-infiltrating lymphocytes after neoadjuvant chemotherapy, *Δ TILs* change in amount of TILs after neoadjuvant chemotherapy

The presence of pre-TILs^high^ was statistically significantly associated with higher pCR rates compared to pre-TILs^low^ in the axilla (58.2 vs. 35.9%, *p *= 0.002), breast (56.7 vs. 31.4%, *p *< 0.001), and combined (49.3 vs. 25.5%, *p *< 0.001). Post-TILs^high^ was conversely associated with lower pCR rates compared to post-TILs^low^ in the axilla (26.1 vs. 47.1%, *p *= 0.01), breast (17.4 vs. 44.8%, *p *= 0.01), and combined (13.0 vs. 37.9%, *p *< 0.001). Additionally, a ΔTILs^decrease^ after NACT was significantly associated with the highest pCR rates compared to ΔTILs^no change^ or ΔTILs^increase^ in the axilla (65.3 vs. 39.9% and 17.9%, *p *< 0.001), breast (69.4 vs. 33.6% and 14.3%, *p *< 0.001), and combined (59.2 vs. 28.7% and 7.1%, *p *< 0.001) (Table 2).

### TILs as a predictor of pathological response

In the univariable analyses, the presence of pre-TILs^high^ was a statistically significant predictor for pCR in the axilla (OR 2.48; 95% CI 1.38–4.46; *p *= 0.002), breast (OR 2.87; 95% CI 1.59–5.18; *p *< 0.001) and combined (OR 2.84; 95% CI 1.56–5.18; *p *< 0.001). On the contrary, post-TILs^high^ was a predictor of non-pCR in the axilla (OR 0.40; 95% CI 0.19–0.82; *p *= 0.01), breast (OR 0.26; 95% CI 0.11–0.59; *p *= 0.001) and combined (OR:0.25; 95% CI:0.10–0.61; *p *= 0.003). The presence of ΔTILs^decrease^ after NACT was associated with a higher likelihood of pCR in the axilla (OR 2.84; 95% CI 1.44–5.99; *p *= 0.003), breast (OR 4.49; 95% CI 2.23–9.03; *p *< 0.001) and combined (OR 3.61; 95% CI 1.84–7.08; *p *< 0.001), compared with ΔTILs^no change^. On contrary, ΔTILs^increase^ was associated with a lower likelihood of pCR in all three groups (Table 3).

**Table 3:** Univariable regression analyses of pre-TILs, post-TILs and ΔTILs in relation to axillary, breast and combined (both breast and axilla) pCR status after neoadjuvant chemotherapy

	*N*	Axillary pCR			Breast pCR			Combined pCR		
		OR	95% CI	*p*-value^a^	OR	95% CI	*p*-value^a^	OR	95% CI	*p*-value^a^
*Pre-TILs*										
Low (< 10%)	153	Ref			Ref			Ref		
High (≥ 10%)	67	2.48	1.38–4.46	0.002	2.87	1.59–5.18	< 0.001	2.84	1.56–5.18	< 0.001
*Post-TILs*										
Low (< 10%)	174	Ref			Ref			Ref		
High (≥ 10%)	46	0.40	0.19–0.82	0.01	0.26	0.11–0.59	0.001	0.25	0.10–0.61	0.003
*Δ TILs*										
No change	143	Ref						Ref		
Increase	28	0.33	0.12–0.91	0.03	0.33	0.11–1.00	0.05	0.19	0.04–0.84	0.03
Decrease	49	2.84	1.44–5.59	0.003	4.49	2.23–9.03	< 0.001	3.61	1.84–7.08	< 0.001
*Age BMI (kg/m*^*2*^*)*										
Per year increase	220	0.97	0.95–1.00	0.03	0.99	0.96–1.01	0.22	0.99	0.97–1.01	0.48
Per unit increase	220	0.94	0.89–0.99	0.04	0.99	0.94–1.05	0.85	0.96	0.90–1.02	0.21
*Tumor subtypes*^b^										
Luminal A&B	77	Ref			Ref			Ref		
HER2 + /Lum	50	3.92	1.71–9.03	0.001	11.8	4.35–32-2	< 0.001	6.66	2.42–18.3	< 0.001
HER2 + /non-Lum	49	24.1	9.31–62-2	< 0.001	40.9	14.0–119	< 0.001	26.8	9.56–75.2	< 0.001
Triple Negative	44	4.95	2.11–11.6	< 0.001	7.45	2.66–20.9	< 0.001	5.52	1.94–15.7	0.001
*Tumor size*^b^										
T1 & T2	151	ref			ref			ref		
T3 & T4	69	0.96	0.54–1.71	0.89	0.70	0.39–1.27	0.24	0.78	0.42–1.44	0.43
*Tumor grade*^b^										
Low (G1 & 2)	132	Ref			Ref			Ref		
High (G3)	86	0.95	0.55–1.64	0.85	1.22	0.70–2.13	0.48	1.19	0.67–2.12	0.56
*Axillary tumor burden*^c^										
< 5 nodes	138	REF			REF			REF		
≥ 5 nodes	82	0.67	0.38–1.17	0.16	0.60	0.34–1.07	0.09	0.64	0.35–1.17	0.15
*NACT completion*^d^										
No (< 75%)	23	ref			ref			ref		
Yes (≥ 75%)	197	1.81	0.71–4.59	*0.21*	2.51	0.90–7.05	0.08	1.86	0.66–5.22	0.24

Other factors (age, BMI, tumor subtypes, size, grade axilla tumor burden and completion of NACT) deemed to have clinically significant relationship with pCR were also included in the univariate regression analyses

^a^Cross tabulations analyses with chi-square test, *p *< 0.05 defined as statistically significant

^b^Denoted pre-NACT status

^c^Approximation of axillary tumor burden at diagnosis based on ultrasound scan assessment without confirmation by histopathology.

^d^NACT completion defined as patient received ≥ 75% of the prescribed intended treatments

*BMI* body mass index, *HER2* + human epidermal growth factor receptor-2 positive, *Lum* Luminal cancer subtype, *NACT* neoadjuvant chemotherapy, *pCR* complete pathological response, *pre-TILs* tumor-infiltrating lymphocytes before neoadjuvant chemotherapy, *post-TILs* tumor-infiltrating lymphocytes after neoadjuvant chemotherapy, *ΔTILs* change in amount of tumor-infiltrating lymphocytes after neoadjuvant chemotherapy

Other factors such as age (OR 0.97; 95% CI 0.95–1.00; *p *= 0.03) and BMI (OR 0.94; 95% CI 0.89–0.99; *p *= 0.04) were statistically significantly associated with lower likelihood of axillary pCR but not with breast or combined pCR. Tumor subtypes including HER2 + /luminal (OR 3.92; 95% CI 1.71–9.03; *p *= 0.001), HER2 + /non-luminal (OR 24.1; 95% CI 9.31–62.2; *p *< 0.001), and TNBC (OR 4.95; 95% CI 2.11–11.6; *p *< 0.001) were statistically significantly associated with higher likelihood of axillary pCR. These same tumor subtypes were also statistically significantly associated with higher likelihood for breast and combined pCR (Table 3).

Separate multivariable analyses were conducted for each of the three TILs categories, adjusted for the factors significant in univariable analysis (age, BMI, and tumor subtype). The presence of pre-TILs^high^ was an independent predictor for pCR in the axilla (OR 2.03; 95% CI 1.02–4.05; *p *= 0.04), breast (OR 2.71; 95% CI 1.31–5.62; *p *= 0.007) and combined (OR 2.70; 95% CI 1.33–5.48; *p *= 0.006). Conversely, post-TILs^high^ was independently predictive of non-pCR in the axilla (OR 0.33; 95% CI 0.14–0.76; *p *= 0.009), breast (OR 0.17; 95% CI 0.07–0.44; *p *< 0.001) and combined (OR 0.20; 95% CI 0.08–0.55; *p *= 0.002). The presence of ΔTILs^decrease^ after NACT was independently predictive of pCR in the breast (OR 4.02; 95% CI 1.71–9.42; *p *= 0.001) and combined (OR 2.99; 95% CI 1.36–6.54; *p *= 0.006) but did not reach statistical significance for axillary response (OR 2.05; 95% CI 0.94–4.47; *p *= 0.07). Conversely, ΔTILs^increase^ was an independent predictor of non-pCR in the axilla (OR 0.25; 95% CI 0.08–0.79; *p *= 0.02), breast (OR 0.22; 95% CI 0.07–0.74; *p *= 0.02) and combined (OR 0.14; 95% CI 0.03–0.67; *p *= 0.01) (Table 4). Another independent predictor was tumor subtype for pCR in the axilla, breast and combined pCR, irrespective of TILs (Table 4).

**Table 4:** Multivariable analyses of TILs-based separately on pre-TILs, post-TILs and ΔTILs, all of which adjusted for clinicopathological factors like age, BMI and tumor subtypes

	*n*	Axillary pCR			Breast pCR			Combined pCR		
		OR	95% CI	*p*-value	OR	95% CI	*p*-value	OR	95% CI	*p*-value
*Pre-TILs*^a,b^										
Low (< 10%)	153	Ref			Ref			Ref		
High (≥ 10%)	67	2.03	1.02–4.05	0.04	2.71	1.31–5.62	0.007	2.70	1.33–5.48	0.006
*Age BMI (kg/m*^*2*^*)*										
Per year increase	220	0.98	0.95–1.01	0.12	1.00	0.97–1.03	0.95	1.00	0.98–1.03	0.77
Per unit increase	220	0.93	0.86–1.00	0.03	1.03	0.95–1.11	0.48	0.97	0.90–1.04	0.39
*Tumor subtypes*^a^										
Luminal (A&B)	77	Ref			Ref			Ref		
HER2 + /Lum	50	3.35	1.42–7.92	0.006	12.7	4.52–35.6	< 0.001	6.28	2.23–17.7	< 0.001
HER2 + /non-Lum	49	21.7	8.21–57.1	< 0.001	40.5	13.5–121.7	< 0.001	24.6	8.62–70.3	< 0.001
Triple-negative	44	4.56	1.87–11.1	< 0.001	5.96	2.07–17.1	< 0.001	4.53	1.55–13.2	0.006

	*N*	Axillary pCR			Breast pCR			Combined pCR		
		OR	95% CI	*p*-value	OR	95% CI	*p*-value	OR	95% CI	*p*-value
*Post-TILs*^a,b^										
Low (< 10%)	174	Ref			Ref			Ref		
High (≥ 10)%)	46	0.33	0.14–0.76	0.009	0.17	0.07–0.44	< 0.001	0.20	0.08–0.55	0.002
*Age BMI (kg/m*^*2*^*)*										
Per year increase	220	0.99	0.95–1.00	0.51	0.99	0.97–1.02	0.62	0.99	0.97–1.03	0.89
Per unit increase	220	0.94	0.88–1.01	0.88	1.05	0.97–1.13	0.21	0.99	0.92–1.06	0.79
*Tumor subtypes*^a^										
Luminal (A&B)	77	Ref			Ref			Ref		
HER2 + /Lum	50	3.76	1.58–8.92	0.003	15.3	5.33–43.7	< 0.001	7.26	2.57–20.5	< 0.001
HER2 + /non-Lum	49	25.2	9.41–67.7	< 0.001	50.0	16.3–153.5	< 0.001	29.2	10.1–84.5	< 0.001
Triple-negative	44	6.93	2.59–15.8	< 0.001	9.81	3.36–28.6	< 0.001	6.96	2.38–20.4	< 0.001

	*N*	Axillary pCR			Breast pCR			Combined pCR		
		OR	95% CI	*p*-value	OR	95% CI	*p*-value	OR	95% CI	*p*-value
*Δ TILs*^a,b^										
No change	143	Ref			Ref			Ref		
Increase	28	0.25	0.08–0.79	0.02	0.22	0.07–0.74	0.02	0.14	0.03–0.67	0.01
Decrease	49	2.05	0.94–4.47	0.07	4.02	1.71–9.42	0.001	2.99	1.36–6.54	0.006
*Age BMI (kg/m*^*2*^*)*										
Per year increase	220	0.98	0.95–1.00	0.11	1.00	0.97–1.03	0.91	1.01	0.98–1.04	0.67
Per unit increase	220	0.93	0.87–1.00	0.06	1.04	0.96–1.12	0.34	0.98	0.91–1.05	0.54
Tumor subtypes*										
Luminal (A&B)	77	Ref			Ref			Ref		
HER2 + /Lum	50	3.70	1.53–8.90	0.004	14.8	5.08–43.0	< 0.001	7.00	2.43–20.1	< 0.001
HER2 + /non-Lum	49	23.5	8.66–63.7	< 0.001	44.8	14.4–139.3	< 0.001	26.6	9.07–77.9	< 0.001
Triple-negative	44	5.33	2.15–13.2	< 0.001	6.79	2.30–20.1	< 0.001	5.18	1.74–15.4	0.003

The adjustment factors were included based on the univariate analyses where they were deemed to have clinically significant impact on pCR outcome after NACT

^a^Denoted as pre-NACT status

^b^Separate multivariate binary logistic regression analyses were conducted with each TILs category where each analysis was adjusted to same variables: age, BMI and tumor subtype

*BMI* body mass index, *HER2* + human epidermal growth factor receptor-2 positive, *Lum* luminal, *NACT* neoadjuvant chemotherapy, *pCR* complete pathological response, *pre-TILs*: tumor-infiltrating lymphocytes before neoadjuvant chemotherapy, *post-TILs* tumor-infiltrating lymphocytes after neoadjuvant chemotherapy, *ΔTILs* change in amount of tumor-infiltrating lymphocytes after neoadjuvant chemotherapy

### Performance characteristics of TILs

In predicting axillary pCR, performance characteristics of pre-TILs, post-TILs and ΔTILs were, respectively, as follows: 42% vs. 13% vs 34% for sensitivity, 78% vs. 73% vs. 87% for specificity, 58% vs. 26% vs. 65% for PPV and 64% vs. 53% vs. 64% for NPV. For predicting breast pCR, performance characteristics of pre-TILs, post-TILs and ΔTILs were, respectively, 44% vs. 9% vs. 40% for sensitivity, 78% vs. 72% vs. 89% for specificity, 43% vs. 17% vs. 69% for PPV and 68% vs. 55% vs. 69% for NPV. And to predict combined pCR, performance characteristics of pre-TILs, post-TILs and ΔTILs were, respectively 46% vs. 8% vs. 40% for sensitivity, 77% vs. 73% vs. 87% for specificity, 49% vs. 13% vs. 59% for PPV and 75% vs. 62% vs. 75% for NPV. All performance characteristics were statistically significant except for post-TILs in predicting axillary pCR (Table 5).

**Table 5:** To assess the diagnostic accuracy of TILs as a predictor of pCR, performance characteristics of pre-TILs, post-TILs and ΔTILs as a predictor were assessed based on sensitivity, specificity, positive predictive value, and negative predictive value using receiver operator characteristics and area-under-curve analyses

	Sensitivity	Specificity	PPV	NPV	Area under curve ROC analysis)	*p*-value	95% CI
*Axillary pCR*							
Pre-TILs	42%	78%	58%	64%	0.59	0.01	0.52–0.67
Post-TILs	13%	73%	26%	53%	0.43	0.07	0.35–0.50
ΔTILs	34%	87%	65%	64%	0.60	0.01	0.53–0.68
*Breast pCR*							
Pre-TILs	44%	78%	43%	68%	0.61	0.01	0.54–0.69
Post-TILs	9%	72%	17%	55%	0.41	0.02	0.33–0.48
ΔTILs	40%	89%	69%	69%	0.64	0.001	0.56–0.72
*Combined pCR*							
Pre-TILs	46%	77%	49%	75%	0.61	0.006	0.53–0.69
Post-TILs	8%	73%	13%	62%	0.41	0.03	0.33–0.48
ΔTILs	40%	87%	59%	75%	0.63	0.001	0.55–0.72

*p *< 0.05 was regarded as statistically significant

*pCR* complete pathological response, *pre-TILs* tumor-infiltrating lymphocytes before neoadjuvant chemotherapy, *post-TILs* tumor-infiltrating lymphocytes after neoadjuvant chemotherapy, *ΔTILs* change in amount of tumor-infiltrating lymphocytes after neoadjuvant chemotherapy, *PPV* positive predictive value, *NPV* negative predictive value, *ROC* receiver operator characteristics analysis

### Axillary pCR and TILs in relation to survival outcome

Since all three categories of TILs were statistically significantly related to pCR in the axilla as well as the breast, Cox regression analyses were conducted based on only pre-TILs and axillary pCR, aligning with the study aims. Additionally, regression analyses were performed using different combinations of pre-TILs and axillary pCR as a single factor and these were grouped into three categories, namely, axillary pCR with pre-TILs^high^, heterogeneous combinations and no axillary pCR with pre-TILs^low^ (Table 6).

**Table 6:** Cox regression analysis of axillary pCR and pre-TILs in relation to breast cancer-free interval and overall survival outcome

Regression analysis based on axillary pCR		Breast cancer-free interval				Overall survival			
		Univariable		Multivariable		Univariable		Multivariable	
		HR (95% CI)	*p*-value	HR (95% CI)	*p*-value	HR (95% CI)	*p*-value	HR (95% CI)	*p*-value
Axillary	No	Ref		Ref		Ref		Ref	
pCR	Yes	0.30 (0.15–0.62)	0.001	0.28 (0.12–0.63)	0.002	0.32 (0.15–0.68)	0.003	0.28 (0.12–0.68)	0.004
Age	continuous	1.01 (0.99–1.04)	0.37	1.00 (0.98–1.03)	0.77	1.04 (1.01–1.06)	0.006	1.03 (1.00–1.06)	0.03
Tumor subtypes	Luminal A&B	Ref		Ref		Ref		Ref	
	HER2 + /Lum	0.47 (0.19–1.17)	0.10	0.60 (0.24–1.52)	0.28	0.51 (0.19–1.40)	0.19	0.74 (0.27–2.06)	0.57
	HER2 + /Non-Lum	0.45 (0.18–1.11)	0.08	1.03 (0.37–2.87)	0.96	0.39 (0.13–1.17)	0.09	1.09 (0.32–3.69)	0.89
	Triple negative	1.67 (0.86–3.26)	0.13	2.30 (1.16–4.56)	0.02	2.16 (1.06–4.43)	0.04	3.2 (1.51–6.82)	0.003
NACT completion	No (< 75%)	Ref		Ref		Ref		Ref	
	Yes (≥ 75%)	0.67 (0.30–1.49)	0.32	0.86 (0.38–1.98)	0.73	0.31 (0.15–0.65)	0.002	0.41 (0.19–0.88)	0.02

Regression analysis based on pre-TILs		Breast cancer-free interval				Overall survival			
		Univariable		Multivariable		Univariable		Multivariable	
		HR (95% CI)	*p*-value	HR (95% CI)	*p*-value	HR (95% CI)	*p*-value	HR (95% CI)	*p*-value
Pre-TILs	Low (< 10%)	Ref		Ref		Ref		Ref	
	High (≥ 10%)	0.50 (0.24–1.04)	0.06	0.46 (0.22–0.96)	0.04	0.71 (0.35–1.45)	0.35	0.73 (0.35–1.55)	0.42
Age	Continuous	1.01 (0.99–1.04)	0.37	1.00 (0.98–1.02)	1.00	1.04 (1.01–1.06)	0.006	1.03 (1.00–1.05)	0.057
Tumor subtypes	Luminal A&B	Ref		Ref		Ref		Ref	
	HER2 + /Lum	0.47 (0.19–1.17)	0.10	0.49 (0.19–1.24)	0.13	0.51 (0.19–1.40)	0.19	0.61 (0.22–1.68)	0.34
	HER2 + /Non-Lum	0.45 (0.18–1.11)	0.08	0.51 (0.20–1.28)	0.15	0.39 (0.13–1.17)	0.09	0.51 (0.17–1.57)	0.24
	Triple negative	1.67 (0.86–3.26)	0.13	1.93 (0.98–3.82	0.06	2.16 (1.06–4.43)	0.04	2.37 (1.12–5.03)	0.02
NACT completion	No (< 75%)	Ref		Ref		Ref		Ref	
	Yes (≥ 75%)	0.67 (0.30–1.49)	0.32	0.74 (0.32–1.69)	0.47	0.31 (0.15–0.65)	0.002	0.40 (0.19–0.87)	0.02

Regression analysis based on different categories of the combinations of axillary and pre-TILs		Breast cancer-free interval				Overall survival			
		Univariable		Multivariable		Univariable		Multivariable	
		HR (95% CI)	*p*-value	HR (95% CI)	*p*-value	HR (95% CI)	*p*-value	HR (95% CI)	*p*-value
Pre-TILs & Axilla pCR combined	Category-1^	Ref		Ref		Ref		Ref	
	Category-2^	0.73 (0.41–1.31)	0.30	0.65 (0.35–1.21)	0.18	0.90 (0.47–1.70)	0.74	0.78 (0.39–1.56)	0.48
	Category-3^	No events #	–	No events #	–	0.09 (0.01–0.69)	0.02	0.09 (0.12–0.72)	0.02
Age	Continuous	1.01 (0.99–1.04)	0.37	1.00 (0.98–1.02)	0.91	1.04 (1.01–1.06)	0.006	1.02 (1.00–1.05)	0.08
Tumor subtypes	Luminal A&B	Ref		Ref		Ref		Ref	
	HER2 + /Lum	0.47 (0.19–1.17)	0.10	0.55 (0.22–1.40)	0.21	0.51 (0.19–1.40)	0.19	0.70 (0.25–1.93)	0.49
	HER2 + /Non-Lum	0.45 (0.18–1.11)	0.08	0.83 (0.32–2.18)	0.71	0.39 (0.13–1.17)	0.09	0.77 (0.24–2.48)	0.67
	Triple negative	1.67 (0.86–3.26)	0.13	2.33 (1.17–4.64)	0.02	2.16 (1.06–4.43)	0.04	3.04 (1.42–6.53)	0.004
NACT completion	No (< 75%)	Ref		Ref		Ref		Ref	
	Yes (≥ 75%)	0.67 (0.30–1.49)	0.32	0.82 (0.36–1.87)	0.63	0.31 (0.15–0.65)	0.002	0.41 (0.19–0.90)	0.03

Multivariate cox regression analyses for each variable: axillary pCR, pre-TILs and in-combinations were all adjusted for age, tumor subtypes and NACT completion. The combined factor of axillary and pre-TILs were divided into three categories^: category-1: no axillary pCR + pre-TILs^low^, category-2: heterogeneous combinations (axillary pCR + pre-TILs^low^ or axillary non- pCR + pre-TILs^high^) and category-3: axillary pCR with pre-TILs^high^

#In the category-3 subgroup with both axillary pCR and pre-TILs^high^, there were no disease recurrence events therefore relevant regression analysis was not able to be performed.

*HER2* + human epidermal growth factor receptor-2 positive, *Lum* luminal, *NACT* neoadjuvant chemotherapy, *pCR* complete pathological response, *pre-TILs* tumor-infiltrating lymphocytes before neoadjuvant chemotherapy, *post-TILs* tumor-infiltrating lymphocytes after neoadjuvant chemotherapy, *ΔTILs* change in amount of tumor-infiltrating lymphocytes after neoadjuvant chemotherapy.

The average duration of follow-up was 4.9 years (range: 0.8 to 9.7 years). During the follow-up period, 4.1% (9/220) developed breast-only recurrences, while 5.5% (12/217) had axillary-only recurrences. Additionally, 22.7% (50/220) developed distant metastases only, of which 82% (41/50) had pre-TILs^low^ tumors. The overall mortality was 18.2% (40/220), including three patients who died from causes unrelated to breast cancer. Of patients who died of breast cancers, 76% (28/37) had low TILs and 24% (9/37) had high TILs, both before and after NACT, respectively. However, noticeably, in the high TILs groups, there were no deaths amongst those patients who had large amount of TILs (> 50%) before and after NACT.

**Fig. 2:**
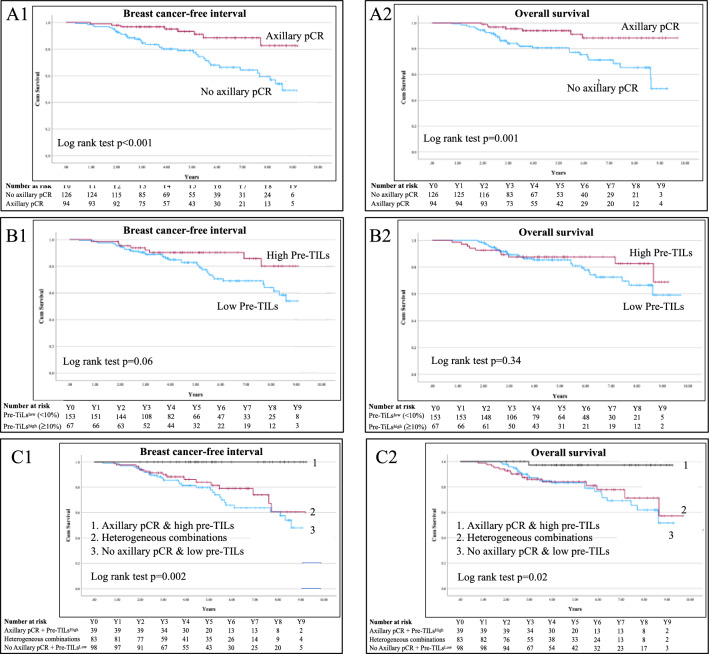
Breast cancer-free interval and overall survival based on axillary pCR (A1 & A2), pre-TILs (B1 & B2) and axillary pCR combined with pre-TILs (C1 & C2). Heterogeneous combinations defined as various combinations of axillary pCR and pre-TILs other than category-1 and -3 for example axillary pCR & low pre-TILs. Statistical Kaplan Meir survival analysis was used with log-rank test *p* < 0.05 defined as statistically significant. Abbreviations: pCR: complete pathological response, pre-TILs: tumor-infiltrating lymphocytes before neoadjuvant chemotherapy

The presence of pre-TILs^high^ was an independent prognostic factor for BCFI (HR 0.46; 95% CI 0.22–0.96; *p *= 0.04) but not for OS (HR 0.73; 95% CI 0.35–1.55; *p *= 0.42). On the other hand, axillary pCR was both a prognostic factor for BCFI (HR 0.28; 95% CI 0.12–0.63; *p *= 0.002) and OS (HR 0.28; 95% CI 0.12–0.68; *p *= 0.004). When considering the combination of axillary pCR wih pre-TILs^high^ as a single factor, it was an independent prognostic factor for OS (HR 0.09; 95% CI 0.12–0.72; *p *= 0.02) compared to the combination of axillary non-pCR with pre-TILs^low^ (Table 5 and Fig. 2). In this study cohort, TNBC subtype and NACT completion were the other two factors statistically significantly associated with BCFI and OS (Table 6).

## Discussion

The main finding in this study was that a large amount of pre-TILs in the primary tumor independently predicted pCR in the axilla, breast, as well as combined axillary and breast responses. Additionally, a subsequent decrease of TILs after NACT was also statistically significantly related to pCR in all groups while an increase in TILs was related to non-pCR instead. However, when considering TILs as a standalone factor, it was not a statistically significant predictor in survival outcome. Nevertheless, when combined with axillary pCR, the results indicated a significantly improved overall survival in the group with pre-TILs^high^ combined with axillary pCR.

Tumor-infiltrating lymphocytes represent the body’s immune response to cancer [22] and have been shown to predict tumor response after NACT [23, 24]. However, there are currently few studies that indicate TILs also predict axillary pCR. In this study, the presence of high pre-TILs represented an immunogenic environment where immune cells were activated against cancer cells thereby leading to pCR both in the breast and axilla. Also, the direction of change where there was a decrease in the amount of TILs was also independently predictive for pCR after NACT. This finding could be explained by the cytotoxic effects of chemotherapy on both cancer and immune cells, especially in pCR patients, resulting in destructions of both cellular cohorts and therefore, decreases in the amount of post-TILs after NACT, seen especially in those with near-CR or pCR [13, 25, 26]. Alternately, a decrease in TILs after NACT could indicate a downregulation of immune response where there is no longer an immunogenic cancer target after NACT [27]. In other words, the immune response mechanism was switched off when it was no longer needed in the event of pCR. The above findings further supported the notion of a dynamic interplay between the immune system and NACT.

Although standalone TILs were shown not related to survival outcome in this study, there were data that suggested high post-TILs was related to improved survival, especially in the HER2 + and TNBC subtypes [12, 28]. Several factors could have limited the impact of TILs on the survival outcome in this study. For example, the small cohort size or short duration of follow-up could explain the lack of statistical significance in the prognostic value of TILs. Further assessments of specific subset of T-cells, such as CD3 + , CD4 + , CD8 + and FOXP3 + in tumor tissues could be useful, as these subsets have been shown to predict survival outcome [29–31]. Another explanation could be that of the definition of high TILs in this study could be too low at only 10%. There are studies that suggested TILs of up to 60% had better survival outcome [14, 15]. In addition, our analyses between TILs and survival outcome could also be limited using the Miller Payne classification, that did not take into the account of axillary tumor response, in comparison with Residual Cancer Burden (RCB) [32]. Since TILs have been shown to be related to axillary response in this study with noticeable performance characteristics, integrating TILs with RCB could provide added prognostic value for breast cancer survival after NACT [33, 34]. Such prognostic information in the presence of residual tumor could further facilitate the identification of suitable patients who could benefit from further systemic therapy.

In terms of surgery, there is data relating safety in breast conservation amongst patients who have achieved good or complete tumor response in the breast [35]. In comparison, the concept of avoiding ALND after NACT has somewhat slower adoption rates amongst surgeons, despite data from several trials have now indicated surgical de-escalation in the axilla is not related to worse survival outcome [36]. Therefore, the potential use of TILs in predicting axillary response and patient selection could further add to the accuracy of selective de-escalation in axillary surgery.

There were limitations associated with this study. As there was no power calculation, this study may be underpowered to detected accurate statistical significances in the relationship between TILs and survival after NACT. Additionally, there could be discordances and inaccuracies in the histopathological assessment of TILs due to paucity of tissues from core biopsy fragments, whereby, any potential discordances could be minimized by having two pathologists to conduct retrospective assessments. However, as TILs were routinely reported according to the international working group guidelines and by certified pathologists at our hospital, we did not feel it was necessary for dual reporting in this study. Nonetheless, further evaluation of TILs by computerized algorithms using artificial intelligence might improve the accuracy [37]. The changes in clinical practice using hot-spot Ki-67 assessments instead of global score occurred during the study period, could have impacts on the accuracy of tumor subtyping, and thereby its association with pCR. However, since we did not observe any statistically significant differences in patient and tumor characteristics between the two different treatment periods, this suggested that there were unlikely significant overall confounding biases despite changes in clinical practice over time. The completion of NACT was defined as 75% of intended therapy as a pragmatic approach. However, it is important to note that there is no consensus on what constitutes an adequate number of NACT cycles that can have a clinically significant impact on breast cancer [38]. Therefore, the chosen cut-off value for NACT completion in this study could lead to biases in the analyses.

In conclusion, our study findings suggested that pre-TILs^high^ was related to axillary pCR and could be used to determine appropriate groups of patients for less aggressive axillary surgery. Pre-TILs^high^ in combination with axillary pCR, as a single factor, could potentially lead to enhanced prognostication of survival outcome in breast cancer patients treated with NACT. Future studies in immune-modulatory biological markers could lead to more accurate predictive and prognostic algorithms for those patients who may benefit from further therapy after NACT.

## Summary statement

The authors *Kian Chin, Amalia H. Landén, Anikó Kovács, Fredrik Wärnberg and Maria Ekholm* have declared no conflict of interests related to this study. Specifically, the author *Per Karlsson* had declared interests that are not related to the study, working on research contracts with PFS Genomics/Exact Sciences and Prelude-Dx as co-inventor for patent applications. *Roger Olofsson Bagge* has also declared interests that are not related to the study, where he has received institutional research grants from Bristol-Myers Squibb (BMS), Endomagnetics Ltd (Endomag), SkyLineDx and NeraCare GmbH, speaker honorarium from Roche, Pfizer and Pierre-Fabre, and has served on advisory boards for Amgen, BD/BARD, Bristol-Myers Squibb (BMS), Cansr.com, Merck Sharp & Dohme (MSD), Novartis, Roche and Sanofi Genzyme, and is a shareholder in SATMEG Ventures AB.

## Data Availability

The data presented in this study are available on request from the corresponding author.
